# “Increasing dopamine synthesis capacity in the dorsal striatum and tonic acetylcholine: implications for the new muscarinic agents in the treatment of schizophrenia”

**DOI:** 10.1038/s41380-026-03758-2

**Published:** 2026-07-09

**Authors:** Darryl Eyles, Zilong Du, Xiaoying Cui, James P. Kesby

**Affiliations:** 1 Queensland Centre for Mental Health Research, Wacol, Brisbane, QLD 4076 Australia; 2 Queensland Brain Institute, University of Queensland, Brisbane, QLD 4072 Australia; 3 Helen Wills Neuroscience Institute, University of California, Berkeley, Berkeley, CA 94720 USA

**Keywords:** Neuroscience, Schizophrenia

The recent positive phase 3 trials of the selective muscarinic 1,4 receptor agonist xanomeline used in combination with trospium (KARXT, Cobenfy^TM^) has rekindled interest in the possibility of therapies for schizophrenia that are not based on dopaminergic receptor antagonism. Targeting acetylcholine (Ach) signalling makes sense as it is involved in numerous behaviours associated with the disorder such as attention, learning, memory, motivation, anxiety, mood, appetite, and reward. Xanomeline was 1st shown to be effective in reducing psychotic symptoms in Alzheimer patients decades ago [1]. This new approach builds on a long legacy of post-mortem studies in patients with schizophrenia showing alterations in muscarinic receptors targeted by Cobenfy^TM^ i.e. M1-4 receptors [2].

We are long-overdue for new drug targets in schizophrenia. However, in the rush to embrace the “*new*”, some commentators have failed to fully understand the “*old*” data showing the tight link between Ach and its control of subcortical dopamine (DA) function [3]. It is now widely agreed that the primary DA abnormality in schizophrenia is pre- rather than post-synaptic. More specifically positron emission tomography studies consistently indicate an increase in presynaptic DA synthesis capacity which is restricted to the dorsal striatum [4]. Revisiting the link between hyperdopaminergia and Ach signalling becomes more urgent given the recent therapeutic success of Cobenfy^TM^ and the prominent distribution of M4 receptors within the dorsal striatum.

Whether Ach levels are altered in patients is unknown. Its low levels and rapid turnover have made it difficult to assess directly in the brains of patients. The best measure we currently have for assessing Ach in human brain is its choline precursor using magnetic resonance spectroscopy. A very recent meta-analysis indicated choline levels were elevated across the brain in patients with the largest effect size being in the basal ganglia [5].

Given the tight regulation of DA release by tonic Ach it makes sense to explore this link particularly in those patients with elevated dorsal striatal DA. However to date, this has not been examined. Therefore, to begin exploring this relationship we have developed a model in rats that selectively elevates tyrosine hydroxylase and GTP cyclohydrolase 1, (the rate limiting enzymes in DA synthesis) in the dorsal striatum. We call this model “*enhanced dopamine in prodromal schizophrenia*” (EDiPs). Details of how this model was constructed and tests of its construct and face validity have been described in detail elsewhere [6–8].

**Fig. 1:**
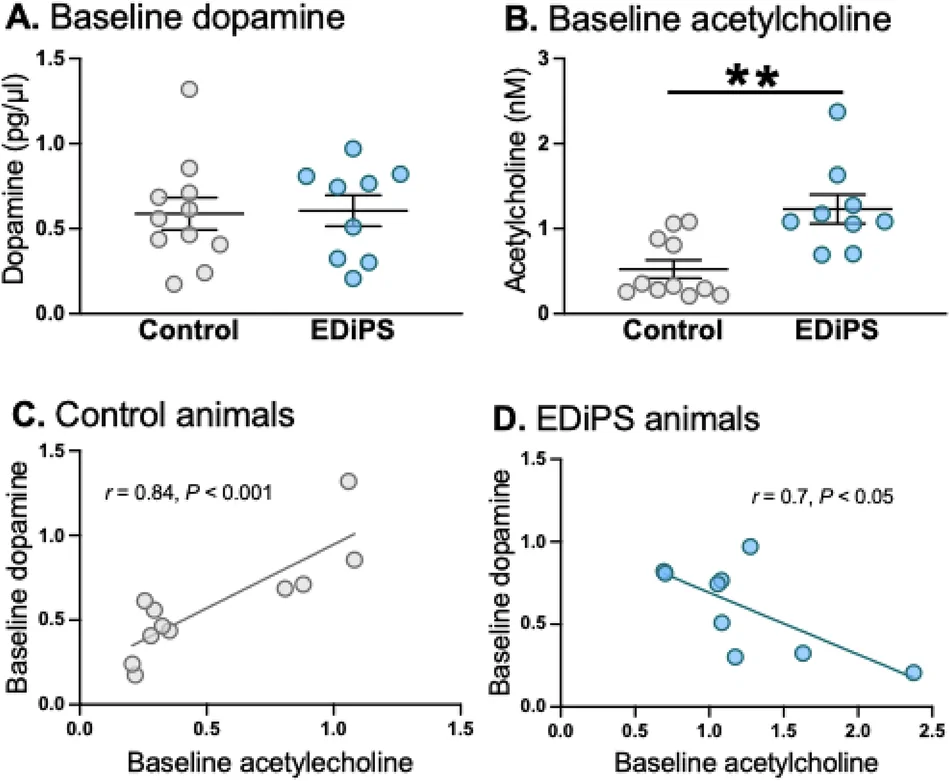
EDiPs induces an increase in baseline acetylcholine in the dorsal striatum. **A** EDiPs induces no alteration to baseline levels of dopamine (*n* = 11 controls *n* = 9 EDiPs). **B** EDiPs increases baseline levels of acetylcholine (*n* = 11 controls *n* = 9 EDiPs) ** *P* < 0.01). **C** Basal levels of acetylcholine and dopamine are positively correlated in control animals (r = 0.84 *P* < 0.001 *n* = 9). **D** Basal levels of acetylcholine and dopamine are negatively correlated in EDiPs animals (r = 0.70 *P* < 0.05 *n* = 9).

Using this model, we conducted unilateral microdialysis in the dorsal striatum of animals that received the active EDiPs construct and those that received the control construct. Baseline DA levels were established using HPLC-ECD [7, 8]. Baseline Ach levels were measured by LC/MS/MS. Here we confirm tonic DA levels are unaltered relative to controls in EDiPs animals (Fig. 1A) as previously established [7, 8]. However, EDiPs led to a doubling of the levels of baseline Ach (Fig. 1B). In the striatum, baseline extracellular Ach is thought to be generated solely by the spontaneous, tonic firing of striatal cholinergic interneurons via action potential dependent vesicular release [9]. Cholinergic interneuron firing is under direct control of cortical glutamatergic inputs, with glutamate release increasing firing rate” [10]. We have previously shown a restricted increase in glutamax (glutamate + its precursor glutamine) in the dorsal striatum of EDiPs animals [7] as well as an increase in cortical glutamatergic terminals again restricted to the dorsal striatum in this model [8]. Whether elevated baseline Ach is a consequence of enhanced tonic firing of cholinergic interneurons in the dorsal striatum of EDiPS animals warrants further investigation.

**Fig. 2:**
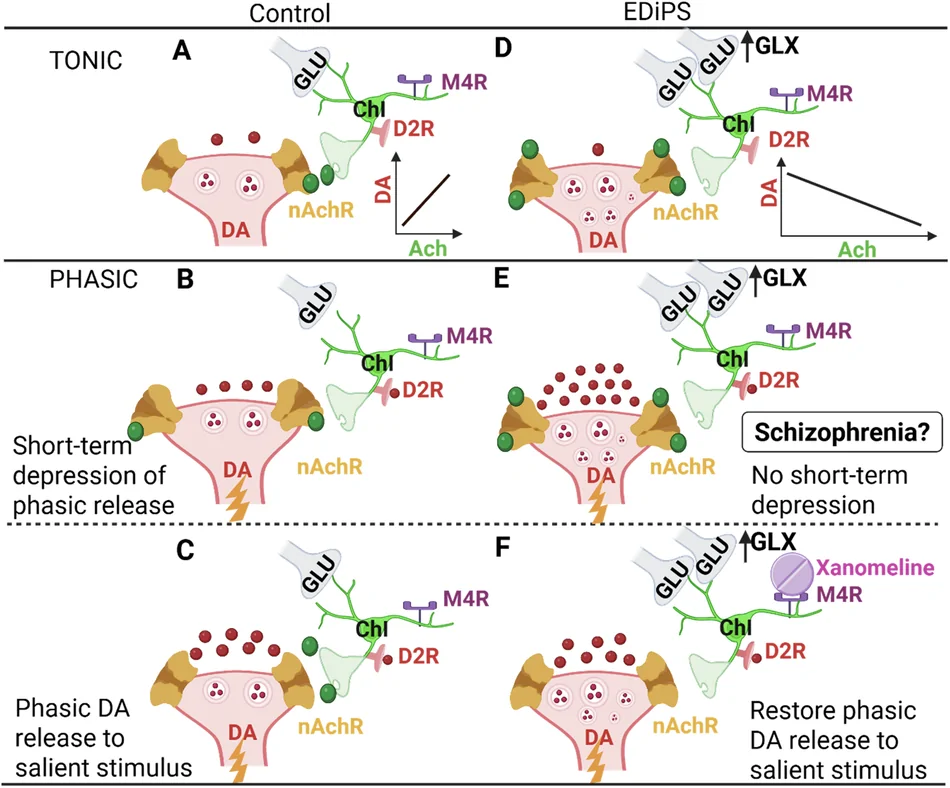
How increased tonic Ach and increased ability to synthesise and release DA may interact in schizophrenia. **A** In a control environment, tonic low levels of Ach stimulates DA release via β2* nicotinic receptors on DA terminals in their open state creating a positive relationship between DA and Ach. **B** When Ach activates β2* nicotinic receptors on DA terminals they undergo a short-term depression of terminal excitability (closed state). This acts to blunt DA release during simultaneous phasic burst-like firing in response to salient stimuli. **C** When these nicotinic receptors are less unoccupied no such restriction on terminal DA release in response to salient stimuli exists, thus tonic Ach levels help to sculpt phasic DA responses to salient stimuli. **D** EDiPs led to a doubling of the levels of baseline Ach possibly due to increased cortical glutamate and glutamatergic terminals in the EDiPs dorsal striatum. Increased tonic Ach would lead to greater β2* nicotinic receptor occupation and a greater probability of desensitisation. Thus, the higher the tonic Ach levels, the lower the amount of background DA. **E** Under phasic dopaminergic burst-firing conditions such a mechanism may be highly relevant to schizophrenia patients who have an enhanced ability to synthesise and release DA. If the β2* nicotinic receptors are persistently desensitised, they will not be able to induce short-term depression induced by on/off activity of Ach binding. This would lead to a greatly enhanced release of DA in response to otherwise normal phasic signal to a salient stimuli. **F** This may in part explain the therapeutic mechanism for Cobenfy^TM^. Activation of M4 autoreceptors on the cholinergic interneurons by xanomeline will reduce tonic Ach release thus restoring tonic cholinergic tone at β2* nicotinic receptors on DA terminals. This would act to normalise DA release to salient phasic stimuli. [Dopamine, red balls; acetylcholine, green balls; Glu, cortical glutamate terminals; D2R, dopamine 2 inhibitory autoreceptors; M4R, muscarinic 4 inhibitory autoreceptors; DA, dopamine; Ach, Acetylcholine; GLX, (glutamate + glutamine); nAchR, β2* nicotinic receptors in open and closed (desensitised) states; ChI, cholinergic interneuron].

As expected, under baseline conditions DA levels in control animals were positively correlated to Ach levels (r = 0.84, *P* < 0.001) (Fig. 1C) consistent with recent studies measuring basal Ach and DA release simultaneously in striatum with genetically encoded fluorescent sensors [i.e., G-protein-coupled receptor-activation-based dopamine and acetylcholine (GRAB_DA_ and GRAB_ACh_)] [11]. However, to our surprise this relationship was reversed in EDiPs animals (r = 0.70, *P* < 0.05) (Fig. 1D). One possible explanation for this may be due to desensitisation of β2* nicotinic receptors on DA terminals. Local Ach in the striatum activates these receptors on DA terminals to induce terminal DA release (Fig. 2A). However, small increases in baseline Ach can rapidly desensitise these nicotinic receptors by inducing conformational changes of the receptor [3, 9]. It may therefore be that the increased baseline Ach in EDiPs animals induces desensitization of β2* nicotinic receptors, thus decreasing the excitability of DA terminals. Thus, a doubling of basal Ach levels as seen in the striatum of EDiPs animals may lower the probability of tonic DA release via such a mechanism (Fig. 2D).

Increases in tonic Ach levels may also have implications for sculpting phasic DA responses to salient stimuli. When Ach activates β2* nicotinic receptors on DA terminals they undergo a short-term depression of terminal excitability [12, 13]. This acts to blunt DA release during phasic burst-like firing in response to salient stimuli and may contribute to the well-described “cholinergic pause” [14] (Fig. 2B). This is also more prominent in the dorsal compared to the ventral striatum [13]. In contrast when these nicotinic receptors are less occupied no such restriction on terminal DA release in response to salient stimuli exists (Fig. 2C). Therefore, tonic Ach levels in the striatum controlled by local Ach interneurons help to sculpt phasic DA response to salient stimuli.

Such a mechanism may be highly relevant to patients with schizophrenia who have an enhanced ability to synthesise and release DA within the DS. If the β2* nicotinic receptors are persistently desensitised due to elevated tonic Ach, on/off receptor occupation may be impaired thus removing short-term depression. The subsequent release of DA in response to a phasic burst-firing response under such conditions may be enhanced due potentially to more vesicular DA available for release (Fig. 2E). Indeed, this is what we have shown for our EDiPs model with increased DA release after electrical stimulation of the median forebrain bundle (as measured by voltammetry) [8].

We speculate that such a situation may exist in the striatum of patients. This may in part explain the therapeutic mechanism for Cobenfy^TM^. The M4 receptors on the cholinergic interneurons act as autoreceptors to block Ach release in response to an agonist. If the increased tonic Ach observed here in our EDiPs model reflects the situation in patients, xanomeline may reduce this to restore cholinergic tone at β2* nicotinic receptors on DA terminals thus normalising DA release to salient phasic stimuli (Fig. 2F). To date there has been no direct examination of xanomeline on both DA and Ach levels in the same animal model. However, an older study showing striatal hyperdopaminergia when the M4 receptor is genetically ablated supports our claim. In this study when amphetamine is used to stimulate DA release the ablation of the M4 receptor leads to a greatly enhanced release of DA [15]. This is presumably due to removal of the inhibitory effects of the M4 receptor on basal Ach release. Under our model the increased basal Ach would desensitise the nicotinic receptors on DA terminals eliminating short-term depression leading to a hyperdopaminergic response to any phasic dopaminergic stimuli or DA-releasing stimulus such as amphetamine.

There are many caveats to such speculation. The actual mechanism for elevated tonic Ach in EDiPs is unknown. Apart from elevated glutamatergic input, cholinergic interneurons are regulated by other factors such as local release of GABA. However, we show GABA levels to be unaltered in EDiPs [7]. There is also a well described inhibition of Ach by DA activating D2 receptors on cholinergic interneurons [16] though this is more likely to be relevant to DA levels achieved from phasic rather than tonic DA release. Ach levels may also be elevated due to a reduction in enzymes such as acetylcholine esterase that is involved in extracellular Ach elimination. It must also be noted that M4 receptors are present on D1 containing medium spiny neurons and thus the possibility also exists for a more direct control of dopaminergic output from the striatum by xanomeline. Finally, increased baseline Ach in the dorsal striatum would likely also activate M1 receptors on striatal medium spiny neurons (MSN)s to potentially affect dopaminergic output. In the striatum both M1 and M4 receptors are on D1 containing MSNs but in D2 MSNs, only M1 receptors are present [17]. Therefore, actions of Ach on M1 receptors will have differential effects based on which dopamine receptor the MSN expresses. For instance, activation of M1 receptors on D2 expressing MSNs could have antipsychotic properties by driving D2 MSN mediated inhibition of D1 output. However, activation of M1 receptors on D1 expressing MSNs would have the opposite effect enhancing D1 MSN mediated output i.e. a likely non-antipsychotic effect.

The therapeutic mechanism for cholinergic agonists in schizophrenia is certainly an avenue of research that should be pursued and is obviously of intense interest given the recent proposal by world leading experts in schizophrenia research for the investigation of “ *muscarinic receptor agonists and positive allosteric modulators in animal models of psychosis: protocol for a systematic review and meta-analysis*” [18]. Though there is much we still do not know regarding the impact of increased DA synthesis and release within the dorsal striatum in schizophrenia patients, the EDiPs model continues to provide important data which informs current and future therapy.
